# Fine-Scale Genetic Structure and Cryptic Associations Reveal Evidence of Kin-Based Sociality in the African Forest Elephant

**DOI:** 10.1371/journal.pone.0088074

**Published:** 2014-02-05

**Authors:** Stephanie G. Schuttler, Jessica A. Philbrick, Kathryn J. Jeffery, Lori S. Eggert

**Affiliations:** 1 Division of Biological Sciences, University of Missouri, Columbia, Missouri, United States of America; 2 Agence Nationale des Parcs Nationaux, Libreville, Gabon; 3 School of Natural Sciences, University of Stirling, Stirling, Scotland; 4 Institut de Recherche en Ecologie Tropicale, Libreville, Gabon; University of Sydney, Australia

## Abstract

Spatial patterns of relatedness within animal populations are important in the evolution of mating and social systems, and have the potential to reveal information on species that are difficult to observe in the wild. This study examines the fine-scale genetic structure and connectivity of groups within African forest elephants, *Loxodonta cyclotis*, which are often difficult to observe due to forest habitat. We tested the hypothesis that genetic similarity will decline with increasing geographic distance, as we expect kin to be in closer proximity, using spatial autocorrelation analyses and Tau K_r_ tests. Associations between individuals were investigated through a non-invasive genetic capture-recapture approach using network models, and were predicted to be more extensive than the small groups found in observational studies, similar to fission-fusion sociality found in African savanna (*Loxodonta africana*) and Asian (*Elephas maximus*) species. Dung samples were collected in Lopé National Park, Gabon in 2008 and 2010 and genotyped at 10 microsatellite loci, genetically sexed, and sequenced at the mitochondrial DNA control region. We conducted analyses on samples collected at three different temporal scales: a day, within six-day sampling sessions, and within each year. Spatial autocorrelation and Tau K_r_ tests revealed genetic structure, but results were weak and inconsistent between sampling sessions. Positive spatial autocorrelation was found in distance classes of 0–5 km, and was strongest for the single day session. Despite weak genetic structure, individuals within groups were significantly more related to each other than to individuals between groups. Social networks revealed some components to have large, extensive groups of up to 22 individuals, and most groups were composed of individuals of the same matriline. Although fine-scale population genetic structure was weak, forest elephants are typically found in groups consisting of kin and based on matrilines, with some individuals having more associates than observed from group sizes alone.

## Introduction

Spatial patterns of relatedness between individuals have important evolutionary consequences, as they influence the formation of mating and social systems within a species [1]. Individuals interact non-randomly, such that mate choice and sex-biased dispersal lead to fine-scale genetic structure. The resulting structure may create opportunities for kin selection [1], [2], affect inbreeding or outbreeding rates [3], or influence local adaptations [4], [5]. Therefore, understanding patterns of underlying genetic relationships offers insight on evolutionary processes within a species, as well as direct applications to conservation and management.

Mammals typically have male-mediated sex-biased dispersal while females are philopatric, creating the potential for matrilocal social groups [6]. One of the best-described mating systems is the breeding group, where one to a few males form permanent or semi–permanent associations with a female group. Groups have high co-ancestry within, but genetic differentiation between due to kin-based relationships within females and shared paternal genes [7]. As geographic distance between individuals increases, genetic structure deteriorates, and kin groups in close spatial proximity to one another will exhibit elevated genetic structure [8], [9].

Species with less rigid or dynamic social systems can still have genetic structure, and even non-social species may exhibit isolation by distance. However, patterns in such species may not be as pronounced as those in species with polygynous breeding groups. For example, species with larger groups such as herd-living ungulates include more members and a larger proportion of the genetic diversity present in the population, weakening genetic patterns [10], [11]. Fission-fusion societies may also have diluted effects, as group sizes change over time. African savanna elephants (*Loxodonta africana*) not only have fission-fusion sociality, but also differ from classic breeding groups because males are not associated with one matrilocal group, but search for mates across the population. Female groups change size over time and space, with some aggregations reaching hundreds of elephants [12], [13]. Matrilocality creates coancestry within and genetic differentiation between core groups, but also cohorts of similar-aged paternal relatives across groups from male gene flow in their prime reproductive years [14].

Here we examine the fine-scale genetic structure of the African forest elephant (*Loxodonta cyclotis*), a species that is not well understood due to the difficulty of conducting studies in its remote and heavily forested habitat. Characteristics of fission-fusion sociality have been detected [15], but observed group sizes are much smaller. Groups appear to be nuclear families typically consisting of an adult female and her dependent calves, with males dispersing [16], [17]. Dung samples collected together revealed relationships between adult females to consist of sisters or half-sisters, and juvenile offspring with occasional instances of more than one reproductive female per group [18]. Associated dung piles included individuals that were significantly more related to each other than to non-associated individuals, but only in one of two populations [18]. Observational studies also indicate social complexity, as some adult females have preferred associations, but individuals are not always found in the same groups or in groups of the same size [19], [20].

The goals of our study were to examine fine-scale genetic structure in a forest elephant population, and investigate the genetic composition and connectivity within and between groups. First, we tested the hypothesis that forest elephants have genetic structure across small scales. We predicted genetic patterns at smaller geographic scales would reveal a decline in genetic similarity with increasing geographic distance. As declining genetic distance with increasing geographic distance could also reflect short-distance dispersal, we also examined associations between individuals using a genetic capture-recapture approach with dung samples. We tested the hypothesis that forest elephants associate with multiple related individuals, consistent with fission-fusion sociality. We predicted that samples collected in a group would be kin-based and include multiple reproductive females, but not always the same individuals. If forest elephants do not have fission-fusion sociality, groups would consist of small nuclear families and individuals would rarely associate with other groups. Therefore dung samples collected together would consist of an adult female and dependent offspring only. If groups reflect aggregations at preferred resources rather than social preferences, genetic composition of dung collected together would be random, and individuals will be largely unrelated. Consistent with results from other elephant species, we predicted that forest elephants would have additional associations outside their nuclear family, and that associations would be based on matrilines and kinship.

## Materials and Methods

### Study Area and Sample Collection

**Figure 1:**
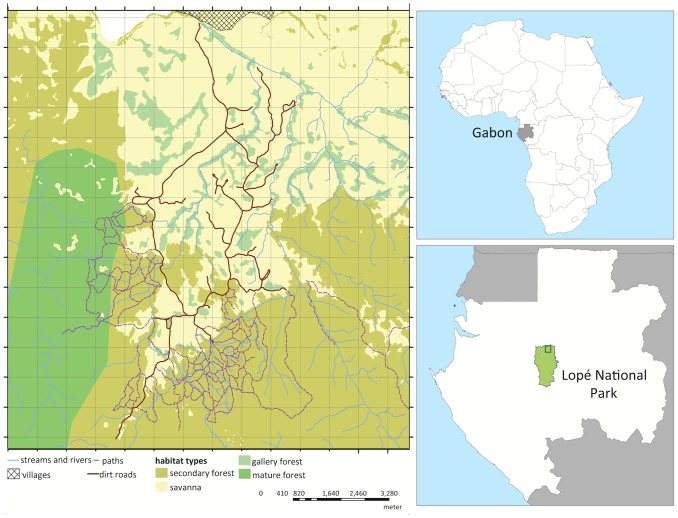
Map showing the SEGC study zone within Lopé National Park, Gabon.

Field research was conducted in the Station d'Etudes des Gorilles et Chimpanzees (SEGC) study zone of Lopé National Park (LNP), Gabon (Figure 1) under permits AR0005/08 and AR0023/09 issued by the Centre National de la Recherche Scientifique et Technologique, the Gabonese government, and Agence Nationale des Parcs Nationaux. Much of LNP is dominated by mature forest, but the northeastern section is characterized by a variety of forest types and savannas [16]. The study zone (approximately 200 km^2^) makes up 3% of the park (4,910 km^2^), but has higher elephant densities with approximately 3.0 elephants/km^2^ [21]. This study was conducted concurrently with an observational study where elephant individuals and groups were searched for throughout the SEGC study zone, individuals were identified, and where possible, dung was collected for genetic analyses from identified groups.

The SEGC study zone was divided into 1.0 km^2^ sections (n = 196) according to Universal Transverse Mercator (UTM) gridlines. Sampling sessions were chosen to capture the range of seasonality. There are two dry seasons, June-August, and a less defined one from December-February. Wet seasons occur in October/November and March/April, although variation occurs between years. Our study included four, five-to-six-day sampling sessions (Table 1); session 1: September 23^rd^–26^th^, 2008 (end of dry season); session 2: October 20^th^–24^th^, 2008 (wet season); session 3: March 21^st^–27^th^, 2010 (beginning of wet season); and session 4: May 10^th^–15^th^, 2010 (end of short wet season, beginning of dry season). Rainfall data were collected daily at SEGC. Sampling sessions were conducted within short time frames to reduce the potential for individuals to move large distances and therefore to obtain dung sample locations that more accurately represent elephant positions in relation to one another.

**Table 1:** Summary of sample collection, rainfall, genotyped samples, age categories, and sexes of unique individuals.

					unique females			unique males		
sampling session	dates	total monthly rainfall (mm)	samples collected	samples genotyped	adult	juvenile	unknown age	adult	juvenile	unknown age
single day	10/24/08	-	40	34	16	2	0	1	3	1
session 1	9/23–9/26/08	134.8	48	39	18	3	3	3	4	-
session 2	10/20–10/24/08	418.5	102	85	25	11	2	8	3	3
session 3	3/21–3/27/10	157.9	63	37	14	3	5	1	4	4
session 4	5/10–5/15/10	115.5	56	51	15	6	1	4	5	1
year 2008	8/12–11/7/08	728.2	239	196	55	16	6	12	14	4
year 2010	2/17–5/10/10	423.5	262	202	57	13	11	6	17	6

For the year analyses, samples were combined with those from a separate observational study (n = 88 from 2008, n = 142 from 2010).

Within each sampling session, between 47–61 1.0 km^2^ sections were searched for fresh (≤24 hours) elephant dung. Sections were chosen randomly each day and teams searched simultaneously in different sections throughout the study zone. Once a section was sampled, the section was not re-sampled during the same session to capture the largest spatial extent. Some samples were collected opportunistically; for example if found on the road while driving to a section. In forested sections, teams searched on and around known elephant trails. In savanna sections, teams searched on elephant paths identified by freshly broken vegetation. If no recent signs were observed, the section was abandoned. Each team included at least one experienced Gabonese field assistant.

Elephant dung can remain visible for months, but changes in appearance and odor make it possible to discern fresh samples [22]. Fresh dung (≤24 hours) was characterized as having sheen, intact boli (unless crushed by elephants or disrupted by insects), and a strong odor (pers. obs). When a fresh sample was found, approximately 10 grams were collected in polypropylene tubes for genetic analyses and GPS coordinates were recorded. If dung piles were intact, up to three bolus circumferences were recorded and averaged to infer the age class of the individual [23]. Samples were boiled for 15 minutes to destroy pathogens and preserved with Queen's College buffer (20% DMSO, 100 mM Tris pH 7.5, 0.25 EDTA, saturated with NaCL; [24]). Samples were stored at room temperature in the dark until the end of each field season (approximately four months), and imported into the US under USDA permit #48529. In the US, samples were stored in the laboratory at 4°C until DNA was extracted, which began within weeks of arrival.

### Laboratory Methods

DNA was extracted using QIAamp Mini Stool Kits (QIAGEN) and modifications in Archie et al. [25] or following the Guanadine Thiocyanate method of Eggert et al. [26]. Extractions took place in a separate room designated for DNA extractions from non-invasive samples to reduce the possibility of contamination.

We genotyped samples using 12 polymorphic microsatellite loci: FH60R, FH94R, FH48R, FH19R, LA6R, LafMS02R [27], FH67, FH126, FH103R [28] FH129 (5′-3′ F-TGGCTAAAATGCCTATCACTCA, R-CCAGCTAAACTAAGTCTGCTCTTTT, [29]), LaT05, [25], and LaT13R [30]. We redesigned primers FH103R (5′-3′ F-GCTGCCACTTCCTACACCTT, R-CCTTTGCTTTTCTAATGAGTCC) and LafMS02R (5′-3′ F-GTCTATCTCCACCCCCTGCT, R-TGTCTGTTGTAAAANTCGCTTG) to shorten fragments. Polymerase chain reactions (PCRs) were performed in a PCR Workstation (Fisher Scientific) with ultraviolet light used between PCRs to decontaminate surfaces.

Samples were amplified in single locus reactions or in four multiplex reactions. Single locus reactions contained 0.5 U AmpliTaq Gold Polymerase (Applied Biosystems), 1X PCR Gold Buffer (Applied Biosystems), 0.4 µM fluorescently labeled forward primer, 0.4 µM unlabeled reverse primer, 2 mM MgCl2, 0.2 mM each dNTP, 1.5 µl 20X BSA, and 3 µl DNA extract in 25 µL reactions. Thermocycling consisted of 95°C for 10 minutes, 45 cycles denaturation at 95°C for 1 minute, primer annealing at locus specific temperatures for 1 minute, and primer extension at 72°C for 1 minute, followed by an extension of 72°C for 10 minutes. Samples amplified at either 58°C or 60°C and were arranged into four multiplex reactions (Table S1). Loci LaT05 and LaT13R were included in all multiplexes because of larger fragment sizes and yielded the same genotypes at both temperatures. Loci LA6R and FH126 had different annealing temperatures from the single to multiplex reactions (54°C to 58°C, and 58°C to 60°C). These loci were tested on two positive controls and 16 samples to confirm that different temperatures resulted in the same genotypes. Multiplex reactions were performed in 8.0 µL volumes containing 4.0 µL Master multiplex mix (QIAGEN), 0.2 µM diluted primer mix, 0.8x BSA, and 1.0–2.5 µL DNA extract. Amplifications were performed with an initial cycle of 95°C for 15 minutes, followed by 40–45 cycles of denaturation at 94°C for 0.5 minutes, primer annealing at 58°C or 60°C for 1.5 minutes, primer extension at 72°C for 1 minute, and a final extension at 60°C for 30 minutes. Each reaction included a positive control to standardize allele scoring and a negative control to detect contamination. PCR products were visualized in 2% agarose gels containing Gel Star (Lonza) to verify amplification.

Fragment analysis was performed using an ABI 3730 DNA Analyzer with Liz 600 size standard (Applied Biosystems) and genotypes were scored in GeneMarker v1.6 (Soft Genetics LLC). Samples were amplified in PCR reactions separately and scored at each locus at least two to three times. Matching heterozygotes from the same sample were scored at least twice and matching homozygotes were scored at least three times to obtain a consensus genotype [31], [32].

We calculated PID_sib_, the power to differentiate between siblings [33], using a subset of 20 genotyped individuals in GenAlEx version 6.41 [34]. We chose the PID_sib_ test over PID_random_ test because it is more conservative and elephants may be found in groups of related individuals [35]. Based on the results, genotypes with at least six loci (PID_sib_ = 0.002) were included in the analyses. PID_sib_ was recalculated once all samples were genotyped, and results did not change. Locus LafMS02R did not amplify reliably and was removed.

Genotyping error rate was calculated using 25 randomly selected samples in Reliotype [36]. We used default settings and 10,000 bootstrap replicates.

We used Dropout [37] to identify genotypes that differed at two or fewer loci, and those genotypes were checked manually. We considered genotypes to represent the same individual if they met the following criteria: (1) at least six matching loci (2) the other loci either did not amplify, or mismatches could be explained by allelic dropout, and (3) one mismatch was allowed if the alleles were difficult to score. Once samples were identified as the same individual, molecular sexes, mitochondrial DNA (mtDNA) haplotypes, and bolus circumferences were compared to ensure accordance. For matches that differed in bolus circumferences, field notes were reviewed to determine if differences were explained by field conditions.

Sex was determined following methods in Munshi-South et al. [38] or Ahlering et al. [39]. A subset of samples was tested for consistency between methods, and because band dropout (*failure to amplify the Y-chromosome*) may occur in bands in samples from males, three independent runs confirmed females, while males were confirmed twice.

We amplified a 627 bp fragment of the mtDNA control region for all individuals identified through unique genotypes using the primers MDL3 and MDL5 [40] or AFDL1, AFDL2, AFDL3, and AFDL4 [41]. Products were sequenced in both directions on an ABI 3730 DNA Analyzer (Applied Biosystems) using the Big Dye Terminator cycle sequencing chemistry. Sequences were aligned and edited in Sequencher 4.5 (Gene Codes Corporation). Haplotypes were identified by at least one base pair difference.

### Data Analysis

We used the average circumferences of three dung boli to establish age classes of juvenile (pre-reproductive) and adult (reproductive) individuals. Eggert et al. [23] considered average bolus circumferences greater than 32 cm to be adults, calibrated from samples based on the age distribution of savanna elephant. As reproductive age has been shown to vary across savanna elephant populations [42], we compared these estimates to samples from known reproductive and pre-reproductive individuals from an accompanying observational study in LNP and adjusted the criteria; samples ≥30 cm were considered adults, while ≤30 cm were juveniles. For individuals with multiple captures and sample averages above and below 30 cm, we averaged all measurements to determine age class. We also looked for evidence of damage (e.g. rain, insects) in field notes, in which case, we relied on measurements of undamaged samples to determine age class.

We used Micro-Checker version 2.2.3 [43] to test for null alleles, stuttering, and large allelic dropout. We used Genepop version 4.0 [44] to calculate observed and expected heterozygosities, allelic diversity for each locus, and to test for deviations from expected heterozygosity values under Hardy-Weinberg equilibrium and for linkage disequilibrium.

To test if genetic similarity declines with increasing geographic distance, we conducted spatial autocorrelation (SA) analyses for adult females using GenAlEx version 6.41 [34]. As samples collected over short time periods resulted in small sample sizes, we tested several scenarios: samples collected together on the same day (single day), each sampling session (week), and combined samples from each year (year) for correlations between genetic and geographic distance. We were only able to conduct one single day analysis due to sample size. For year analyses, samples collected during the spatial genetics sampling were combined with samples collected during the observational study, which were collected differently. In the observational study, circuits by vehicle were conducted almost daily for four-month periods after sunrise and before sunset to search for forest elephant groups and individuals. Circuits covered all roads within the study area (Figure 1), and directions and starting locations were altered to reduce bias in sampling areas. Elephants were observed in savanna habitats, and areas were searched for dung samples after individuals left. Although samples were not searched for randomly in sections, samples were collected throughout the study zone.

Spatial autocorrelation analyses are based on a single location per individual. For repeat captures of individuals within a sampling session, we calculated a midpoint between two locations, or created centroids using minimum convex polygons with Hawth's tools v3.2 (www.spatialecology.com/htools/) in ArcGIS 9.2 (ESRI, Redlands, CA) with more than two locations. Each SA was run with 9,999 permutations and bootstraps. Genetic distances were calculated from genotypes and converted to the autocorrelation coefficient *r*, which although not the same as, yields similar estimates to Queller & Goodnight's *R* [45], [46].

Spatial autocorrelation analyses are influenced by the distance class used [47]. We searched for biologically meaningful classes that would not be overly influenced by short-term movements of elephants during sampling sessions, or by distances between locations of recaptured individuals. We explored telemetry data used in Momont [20] from four adult female elephants in LNP. These elephants moved 2.8–4.4 km every 12 hours [20], but did not displace this distance. Therefore, we examined displacement distances of individuals by selecting one six-day period (the average number of days in week sampling sessions) from each month per individual, and recorded the longest distance between two location points. Average displacement was 4.8±2.3 km with a range of 2.8–7.2 km. Therefore, we chose distance classes of 5.0 km.

Significance in SA can be detected if (1) *r* exceeds the upper and lower bounds of the 95% confidence interval generated from random permutations, or (2) the 95% error about *r* generated from bootstrap tests does not intercept the x-axis at *r* = 0. The latter is more conservative and will favor the null hypothesis more frequently [47]. When positive significant genetic structure is found, *r* will decrease as distance size classes increase. The first distance class where *r* is not significant designates the spatial extent of genetic structure in the population [47]. This can depend on the size of the distance class, which is chosen by the user. To overcome this, we conducted analyses that plotted pairwise genetic distances against increasing inclusive distance classes (999 bootstraps) of 1.0 km intervals to determine the distance class at which *r* was no longer significant [47].

No relatedness estimator outperforms others, and an estimator is data dependent [48]. We used Coancestry version 1.0 [49] to perform Monte Carlo simulations that calculated correlation coefficients between seven relatedness estimators and the values of known relatedness categories generated through simulations using observed allele frequencies and missing genotype rates. We simulated eight relationship categories of 100 dyads, with 100 reference individuals, and 1,000 bootstraps. We chose the Queller-Goodnight moment estimator [45] because it resulted in a strong correlation between true and estimated values (*r* = 0.911). We calculated pairwise relatedness (*R*) in Relatedness version 5.0.8 [45] using the bias correction. To test the effectiveness of this estimator, we used eight known mother-calf pairs whose samples were collected during the observational study. Average pairwise relatedness of mother-calf pairs was 0.490±0.083, consistent with expectations.

To assess the consequences of using centroids for recaptured locations from the same individual, we conducted permutation tests between matrices of dyads that compared spatial proximity with relatedness, but allowed for nonindependent data points. We used Hemelrijk's Tau K_r_ test [50] in Matrixtester (www.rug.nl/fmns-research/beso/_people/hemelrijk) with 10,000 permutations. Matrixtester allows for matrices containing dyads of ≤100 individuals, and therefore the year analyses for 2008 and 2010 were excluded because recaptures increased matrix size.

To understand how kin are positioned in space, we calculated average distances between related adult females in week and single day sessions. Dyads with a relatedness value of at least 0.2 had their Euclidean distances measured and averaged in ArcGIS 9.2. We chose 0.2 to ensure the following relationships would be captured; mother-daughter (*R* = 0.5), full siblings (*R* = 0.5), half siblings (*R* = 0.25), and grandmother-granddaughter (*R* = 0.25), as the mean expected relatedness value on average is 0.25, but can be lower or higher. Mitochondrial DNA haplotypes were mapped into ArcGIS 9.2 to examine the distribution of matrilines.

To investigate associations between individuals, we examined relatedness within and between groups of dung samples, and created network models from samples collected in groups. Samples were considered to be from the same group if they were collected on the same day, within 250 m of each other for each pair of samples, and were of the same freshness. For most samples, identifying groups was apparent, as it was rare to find numerous dung piles that were less than 24 hours old in the same area and of varying freshness. To be conservative, if there was any doubt that dung samples belonged to the same group, they were assigned to different groups.

We tested if individuals within a group were more related compared to individuals from other groups using permutation tests in Perm version 1.0 with 10,000 permutations and 10 iterations [51]. We tested adult females in groups, and all individuals found together. Additionally, we tested for differences in mean pairwise relatedness between groups of male and female dyads in Coancestry using 10,000 bootstraps.

We created network models from the genetic information from dung samples collected together, excluding samples found solitarily, and therefore not associated with any group. Networks were created in Ucinet version 6.403 [52] and NetDraw version 2.120 [53]. We reported the number of nodes (individuals), edges (ties between individuals if samples were detected in groups), and the number of components (nodes connected to each other and not connected to the rest of the network). Edges were weighted by relatedness and mitochondrial haplotypes were added as an attribute. We calculated average relatedness for each component using Relatedness version 5.0.8.

## Results

We collected 501 dung samples and identified 89 unique adult females, 22 juvenile females, 16 females of unknown age, 18 adult males, 22 juvenile males, and 10 males of unknown age from genotypes. On average, individuals were recaptured 2.249 times with a range of 0–19 recaptures. Between 2008 and 2010, 23 adult females were re-sampled.

After applying a standard Bonferonni correction for multiple tests, all loci except LaT05 conformed to Hardy-Weinberg equilibrium expectations. This locus showed evidence of large allelic dropout and null alleles, and because it did not amplify consistently, removing it from analyses did not affect the number of genotyped individuals. The average number of alleles per locus was 12±3.9 and the mean observed heterozygosity was 0.820±0.081 (Table S1). The average expected overall reliability after replication was 0.995.

Mitochondrial haplotype diversity was 0.805±0.017 and nucleotide diversity averaged over all loci was 0.008±0.004. Ten haplotypes were identified, all of which differed from those previously reported of the same length (GenBank accession #KF938592-938601). One haplotype (Lope10) was unique to males.

**Figure 2:**
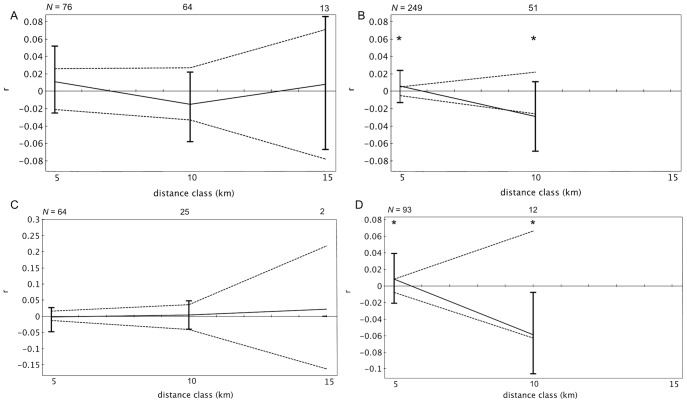
Spatial autocorrelation correlograms for adult female pairs, by week. (A) Represents session 1, (B) 2, (C) 3, and (D) 4. Significant distance classes are designated with an asterisk (*). Dotted lines represent the upper and lower bounds of the 95% confidence interval generated from random permutations, while bars represent 95% error generated from bootstrap tests.

**Figure 3:**
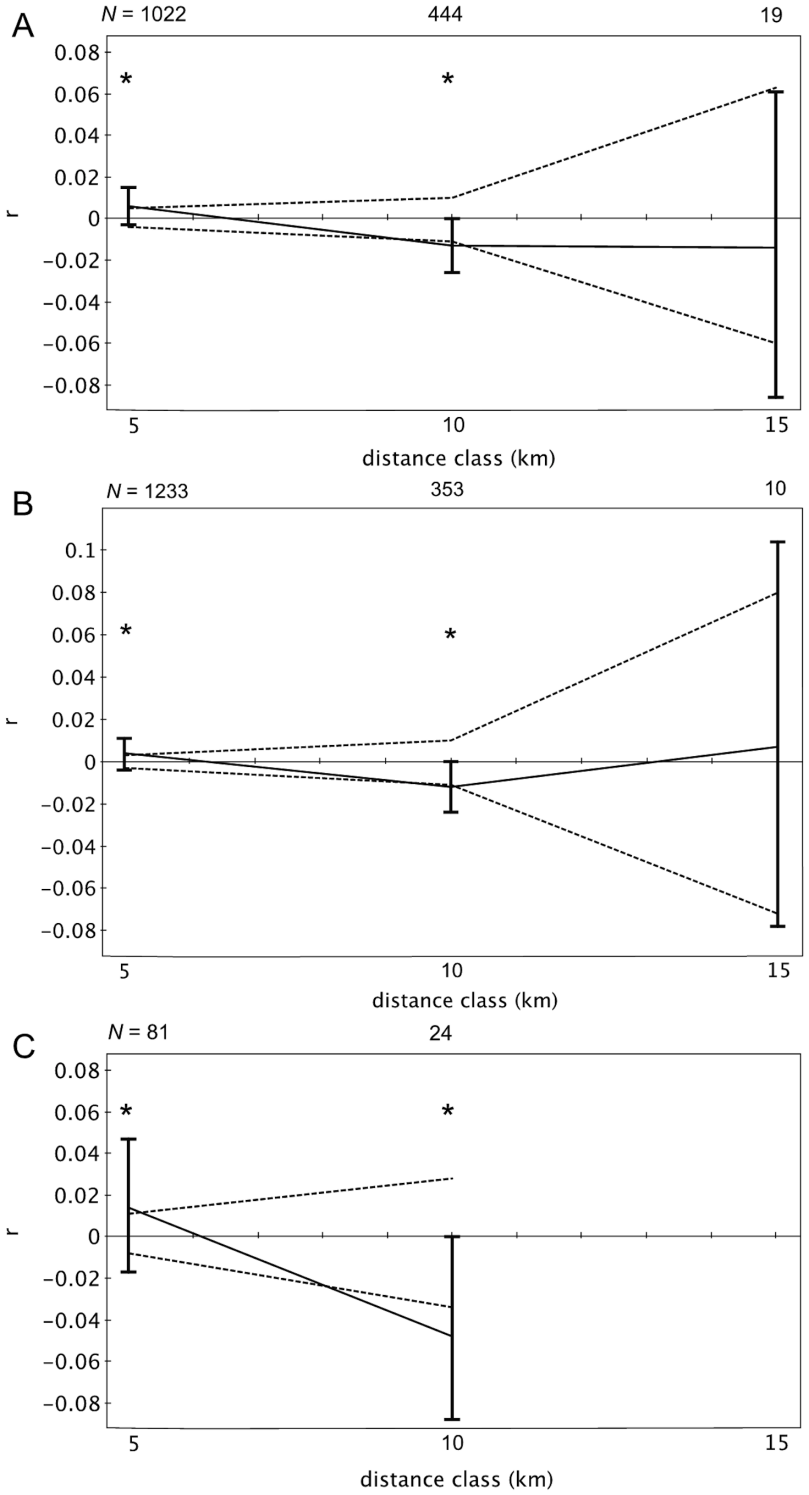
Spatial autocorrelation correlograms for adult female pairs, by year and day. (A) Represents correlogram from year 2008, (B) 2010, and (C) a single day. Significant distance classes are designated with an asterisk (*) and based on 95% confidence intervals from permutation analysis. Dotted lines represent the upper and lower bounds of the 95% confidence interval generated from random permutations, while bars represent 95% error generated from bootstrap tests.

Sessions 2, 4, the single day analysis, and both years revealed significant positive genetic structure at 0–5 km and negative structure at 5–10 km for adult females (Figures 2, 3). Spatial autocorrelation analyses with inclusive distance classes revealed significance up to 5.0 km distances in sessions 2, 4, the single day, 2008, and 2010 (Table 2). However, preceding distance classes were not necessarily significant. No autocorrelation coefficients were significant in sessions 1 and 3 (Figure 2). The Tau K*_r_* tests revealed slightly different results with the significance of non-independent data between spatial proximity and relatedness occurring in session 1 (Tau K*_r_* = −0.106, p = 0.003), 4 (Tau K*_r_* = −0.101, p = 0.006), the single day (Tau K*_r_* = −0.121, p = 0.003), approaching significance in session 2 (Tau K*_r_* = −0.029, p = 0.055), but none in session 3 (Tau K*_r_* = −0.049, p = 0.180).

**Table 2:** Summary of *r* coefficients from autocorrelation analyses with inclusive distance classes for adult female forest elephants in Lopé National Park, Gabon.

	distance class (km)													
sampling session	0 to 1	0 to 2	0 to 3	0 to 4	0 to 5	0 to 6	0 to 7	0 to 8	0 to 9	0 to 10	0 to 11	0 to 12	0 to 13	0 to 14
session 1 AF	0.083	0.044	0.013	0.022	0.011	0.015	0.006	0.002	0.001	−0.001	0.000	0.000	-	-
session 2 AF	**0.059**	**0.016**	0.008	0.004	**0.006**	−0.001	0.001	0.000	-	-	-	-	-	-
session 3 AF	−0.011	−0.004	−0.016	0.000	−0.002	0.002	0.003	−0.002	−0.001	−0.001	0.000	-	-	-
session 4 AF	0.033	0.029	0.015	0.002	**0.008**	0.003	0.003	0.000	-	-	-	-	-	-
single day	**0.075**	**0.025**	**0.013**	**0.014**	**0.014**	0.007	0.002	−0.001	-	-	-	-	-	-
year 2008 AF	**0.026**	0.005	0.003	0.004	**0.006**	0.002	0.000	−0.001	0.000	0.000	0.000	0.000	0.000	0.000
year 2010 AF	**0.029**	**0.012**	0.004	**0.006**	**0.004**	0.001	0.000	0.000	0.000	0.000	0.000	-	-	-

Significant *r* coefficients from one-tailed test for positive autocorrelation P values are in bold type.

The mean distance between adult females with a relatedness value of ≥0.2 was 2.984±2.105 km (session 1), 2.208±1.709 km (session 2), 2.599±0.747 km (session 3), and 1.688±1.775 (session 4). The single day had a smaller mean distance of 0.823±0.548 km. The north-south sampling extent of the single day analysis was slightly smaller than the regular sampling sessions (9 km compared to a maximum of 13–14 km); however the east-west spatial extent was the same (7 km compared to 7–8 km). In all sessions, we found dyads that were closer to each other in distance, but less closely related.

We detected ten mtDNA haplotypes. Lope7 was the most common, but individuals with different haplotypes were found in close proximity (<1.0 km), and overall no spatial segregation was observed. The single day session yielded the same pattern, with samples collected <100 m from each other having different haplotypes.

We detected 70 groups and 26 included more than one adult female (Table S2). Individuals within groups were significantly more related to each other than to individuals from distant groups for adult females (*R* = 0.236±0.187, p<0.001) and all individuals (*R* = 0.255±0.194, p<0.001). In 84.6% of groups (22 of 26), adult females had the same mitochondrial haplotype, and in 82.9% of groups (58 of 70), all individuals shared the same mitochondrial haplotype (Table S2). Consistent with male sex-biased dispersal, females had a significantly higher mean pairwise relatedness than did males (males: *R* = −0.045±0.146; females: *R* = −0.007±0.152, p<0.05).

**Figure 4:**
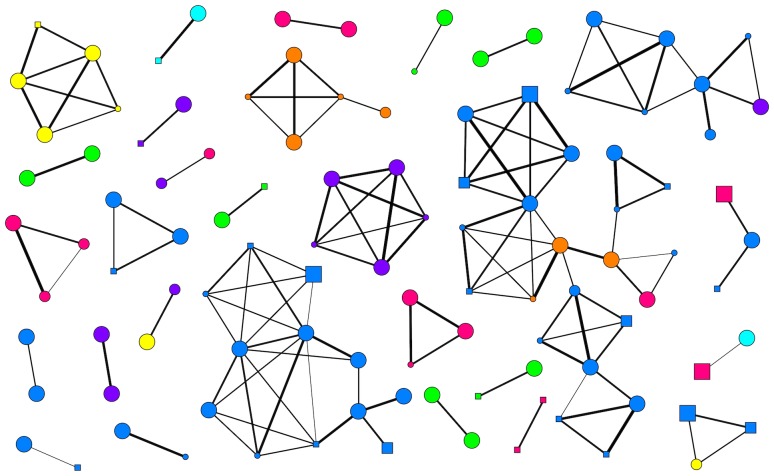
Network constructed from dung sample group data using all individuals. Dung samples that were collected outside of groups were not included. Nodes represent individuals and edges indicate individuals whose dung was collected as part of the group. Squares represent males, while circles represent females. The size of the node reflects the age category; adults are the largest, unknown ages are of medium size, and juveniles are the smallest. Colors represent mitochondrial DNA haplotype; pink, Lope1; orange, Lope3; yellow, Lope4; green, Lope5; aqua, Lope6; blue, Lope7; purple, Lope9. Edges are weighted according to relatedness; those with thicker lines representing more closely related dyads.

The network included 106 individuals with 28 components and 83 edges (Figure 4). The largest component had 22 individuals. Eighteen of 131 dyads were detected two or more times together (13.7%), with six being the largest number of times two individuals were detected together. Average relatedness of network components was 0.155±0.197 for all individuals, and 0.2058±0.167 when including only adult females. Twenty-one components consisted of individuals that shared the same haplotype (75.0%).

## Discussion

This study revealed fine-scale genetic structure, albeit weak, and evidence of potentially complex, kin-based groups using a novel network model genetic approach derived from non-invasive dung sampling in African forest elephants. We tested the hypothesis that genetic similarity decreases with increasing geographic distance for two types of spatial genetic patterns; spatial autocorrelation (SA) analyses in which distance was separated into classes of 5 km, and the Tau K_r_ test, which tests for the relationship between relatedness and distance, but accounts for non-independent repeat locations. Spatial autocorrelation analyses revealed that adult females are more closely related than expected by chance from 0–5 km in two of the four week sampling sessions, samples collected over four-months in 2008 and 2010, and in the single-day analysis. No genetic structure was found in session 1, but there was a significant correlation between distance and relatedness in the Tau K_r_ test. Alternatively, significant genetic structure was detected in sampling session 2, but not in the Tau K_r_ test, although it approached significance (p = 0.055). Conflicting results between the two methods may be due to this test's use of recaptures and not midpoints.

Significant SA coefficients were 0.014 or lower, and for inclusive distance classes, 0.083 or less within the first distance class. Although significance was detected, compared to other mammals (0.04, black rhinoceros *Diceros bicornis bicornis* [54]; 0.07, Eurasian badgers, *Meles meles* [55]; 0.08, tree-roosting bats [9]; 0.12 in Australian bushrats, *Rattus fuscipes* [47]), these values are low. The weaker genetic structure and inconsistent results between sampling sessions may be the result of using larger distance classes, which were necessary given the mobility of elephants and recaptures of samples. This pooling of dyads into more inclusive distance classes may have biased SA coefficients downward. Results of the SA analyses with inclusive distance classes supports this (except session 3), as the autocorrelation coefficients in the smaller distance class of 0–1 km are higher than those in 0–5 km from regular SAs.

Interestingly, while SA analyses revealed significant positive genetic structure in the 0–5 km distance class for most sampling sessions, previous classes in the inclusive SA analyses were not necessarily significant. Related adult females were found at distances of less than one kilometer, and again at approximately five kilometers. As elephants have the ability to move over short time periods, the genetic structure may be diluted in some distance classes because the actual locations of individuals in relation to one another at the exact same time could not be captured. The dung samples are collected within a 24-hr window and therefore locations represent one position for the individual. Distance classes were pooled at larger intervals of five kilometers to account for this approximation. When distance classes are at a finer scale (inclusive SA analyses), individuals may not be placed in the distance class that more accurately represents their true positions relative to one another at that time. The only session to yield significant positive genetic structure with all analyses, and in all of the inclusive distance classes up to five kilometers, was the single day session. This suggests that the time involved in sampling is likely an important factor in capturing fine-scale spatial genetic patterns of forest elephants. The single day analysis includes recaptures, but likely reduced the effects of movements because of the shorter time scale. However, reducing sampling time also limits sample size. To examine these effects, we increased sample size by using data from dung samples collected for each year. Both 2008 and 2010 yielded similar patterns with significant positive genetic structure in the 0–5 km distance class and negative in the 5–10 km class. Future studies should therefore concentrate on increasing sampling effort over shorter time periods.

When looking at the spatial patterns of related dyads (those with a relatedness value of ≥0.2), related adult females were found within several kilometers of each other. However, it was not uncommon to find more geographically distant dyads more closely related than individuals that were closer together, even for samples collected on the same day. There was no spatial structure associated with mitochondrial haplotypes, and based on dung locations and dates, individuals appeared to tolerate others with different haplotypes in close proximity. This may explain the weak genetic structure from the SA analyses and inconsistent results between sampling sessions. Although we detected a signal of positive genetic structure within five kilometers, it is likely diluted by the presence of unrelated individuals in the area. However, consistent with evidence of male-biased dispersal that typically occurs in mammals, females had a significantly higher mean pairwise relatedness than males in the population.

The SEGC study zone differs from most forest elephant habitat because it is a savanna-forest mosaic and also has slightly higher elephant densities [21]. Group sizes do not differ between forested and savanna habitats, and are comparable in size and composition to those found in other populations, with an adult female and her calves being the most common group type [16]. In Loango National Park, a protected area with similar habitat and elephant densities, mean relatedness in associated dung piles was not significantly higher than non-associated piles, which contradicts the pattern found in another population tested in that same study, and also results from this study [18]. This could be due to high elephant densities there, which could increase the chance of sampling different groups using the same area or seasonal migrations as unrelated groups would be in closer proximity when individuals migrate into the area. However, Schuttler et al. [56] found no evidence of seasonal migrations in the home ranges of forest elephants in Loango. White [57] demonstrated that elephant dung was seasonally correlated with the ripening of *Sacoglottis gabonensis*, with dung rates highest in September – November. Our study found genetic structure through SA in only one of the two of the sampling sessions that occurred in the months with both the most and fewest number of dung piles. Therefore, genetic structure can be detected, even when densities are high, however, higher densities also have the potential to dilute patterns, necessitating the importance of looking at individual association patterns.

We also did not find any relationship between rainfall and the results of spatial genetic analyses. Session 2 occurred during the month with the most rainfall, and we found significant SA, and an almost significant negative relationship between relatedness and spatial proximity in the Tau K_r_ test. In contrast, session 3 had the second highest amount of rainfall, yet no spatial genetic structure was detected.

Other factors possibly contributing to inconsistent results may include social structure, poaching, and sample size. Evidence is mounting that forest elephants have fission-fusion sociality, where group sizes and composition change over time [15]. Sessions where genetic structure is detected could reflect periods when individuals group together. Poaching has had a severe impact on forest elephant populations [58] and may also influence social structure. Gobush et al. [29] found that non-kin grouped together in poached populations of savanna elephants. We found groups of associated dung piles to consist largely of individuals of the same matriline, therefore this is unlikely true for the LNP population. Another aspect is the small sample sizes in SA analyses. Although sampling sessions yielded adequate sample sizes of dung samples, removing juveniles, males, samples that would not reliably amplify using the PCR, and recaptures of individuals reduced sample sizes.

Despite weak genetic structure, we found relatedness in dung samples collected as groups to be consistent with family group expectations. Individuals within groups were significantly more related to each other than to individuals from other groups, and average pairwise relatedness between adult female elephants (0.236) was comparable to family groups in savanna elephants (0.150, 35,0.234, [59]). We linked samples over time using network models to investigate associations at the population level and found several components with more complex associations, despite behavioral studies revealing an adult female and dependent calves as the most common group type [16]. The largest component contained 22 individuals and six larger components (5–12 individuals) were found, with a mean of 3.786 for all components, and a median of two. Additionally, 75% of components consisted of individuals that shared a mtDNA haplotype and the average relatedness of individuals within components was 0.155, suggesting most associations are kin-based. However, 25% of associations were between different matrilines, which could represent group formation independent of kin [29], or cases where unrelated individuals used the same resources. Some individuals therefore had a larger number of associations than what is reflected from group sizes alone, and surprisingly most were based on the same matriline despite high haplotype diversity in the area and the close proximity between dung samples of different haplotypes. However, many components were small and consistent with group sizes from behavioral observations. Group sizes from dung collection may be underestimated as not all members may defecate, and not all samples collected were successfully genotyped. Therefore further research is still needed to address how common larger components are in forest elephant society.

Tracking associations from dung found together, combined with fine-scale spatial genetic sampling, allows for information about elephant sociality that cannot be gained from observations alone. Observational sampling is biased towards diurnal observations, as only groups visible or active during the day are observed. Using acoustic sampling, Wrege et al. [60] found that 79% of forest elephant visitations occurred at night. Although genetic methods have caveats including under-sampling groups, they can capture diurnal and nocturnal associations, as well as cryptic associations in forested habitat. Our network model created from the genetic information from non-invasive dung sampling revealed that individuals can associate with a larger number of individuals than what is reflected through group sizes, and that individuals are not always found with the same associates, which is consistent with the expectations of fission-fusion sociality. Although more information is needed to understand the full social repertoire of forest elephants, our results demonstrate that associations can be larger than what is observed in group sizes alone, and that forest elephants more often associate with individuals of the same matriline, even in genetically diverse populations. By combining results from observational studies to the association patterns detected with dung, we can get a clearer picture of forest elephant sociality.

## Supporting Information

Table S1 **Genetic diversity values for elephants at Lopé National Park, Gabon.** N_a_ = allelic diversity, H_e_ = expected and H_o_ = observed heterozygosity. Multiplexes 1 and 4 had an annealing temperature of 60°C, while 2 and 3 were at 58°C.
(DOCX)

Table S2 **Group composition, mitochondrial haplotypes, average pairwise relatedness (*R*) and 95% confidence intervals (CI) within groups.** A–adult, J–juvenile, U–unknown age category, F–female, and M–male.
(DOCX)

## References

[1] Johnstone RA, Cant MA (2008) Sex differences in dispersal and the evolution of helping and harming. The American Naturalist 172: 318–330. 10.1086/589899 18662138

[2] Woxvold IA, Adcock GJ, Mulder RA (2006) Fine-scale genetic structure and dispersal in cooperatively breeding apostlebirds. Molecular Ecology 15: 3139–3146. 16968260 10.1111/j.1365-294X.2006.03009.x

[3] Hazlitt SL, Sigg DP, Eldridge MDB, Goldizen AW (2006) Restricted mating dispersal and strong breeding group structure in a mid-sized marsupial mammal (*Petrogale penicillata*). Molecular Ecology 15: 2997–3007. 16911216 10.1111/j.1365-294X.2006.02985.x

[4] Garant D, Kruuk LEB, Wilkin TA, McCleery RH, Sheldon BC (2005) Evolution driven by differential dispersal within a wild bird population. Nature 433: 60–65. 15635409 10.1038/nature03051

[5] Postma E, van Noordwijk AJ (2005) Gene flow maintains a large genetic difference in clutch size at a small spatial scale. Nature 433: 65–68. 15635410 10.1038/nature03083

[6] Greenwood PJ (1980) Mating systems, philopatry and dispersal in birds and mammals. Animal Behaviour 28: 1140–1162.

[7] Storz JF (1999) Genetic consequences of mammalian social structure. Journal of Mammalogy 80: 553–569.

[8] Hazlitt SL, Eldridge MDB, Goldizen AW (2004) Fine-scale spatial genetic correlation analyses reveal strong female philopatry within a brush-tailed rock-wallaby colony in southeast Queensland. Molecular Ecology 13: 3621–3632. 15548278 10.1111/j.1365-294X.2004.02342.x

[9] Rossiter SJ, Zubaid A, Mohd-Adnan A, Struebig MJ, Kunz TH, et al (2012) Social organization and genetic structure: insights from codistributed bat populations. Molecular Ecology 21: 647–661. 22168272 10.1111/j.1365-294X.2011.05391.x

[10] Robinson SJ, Samuel MD, Lopez DL, Shelton P (2012) The walk is never random: subtle landscape effects shape gene flow in a continuous white-tailed deer population in the Midwestern United States. Molecular Ecology 21: 4190–4205. 22882236 10.1111/j.1365-294X.2012.05681.x

[11] Nussey DH, Coltman DW, Coulson T, Kruuk LEB, Donald A, et al (2005) Rapidly declining fine-scale spatial genetic structure in female red deer. Molecular Ecology 14: 3395–3405. 16156811 10.1111/j.1365-294X.2005.02692.x

[12] Wittemyer G, Getz WM (2007) Hierarchical dominance structure and social organization in African elephants, *Loxodonta africana* . Animal Behaviour 73: 671–681.

[13] Moss CJ (1988) Elephant memories. Chicago: University of Chicago Press.

[14] Archie EA, Maldonado JE, Hollister-Smith JA, Poole JH, Moss CJ, et al.. (2008) Fine-scale population genetic structure in a fission-fusion society. Molecular Ecology: 2666–2679. 10.1111/j.1365-294X.2008.03797.x 18466226

[15] Fishlock V, Lee PC (2012) Forest elephants: fission–fusion and social arenas. Animal Behaviour 85: 357–363.

[16] White LJT, Tutin CG, Fernandez M (1993) Group composition and diet of forest elephants, *Loxodonta africana cyclotis* Matschie 1900, in the Lopé Reserve, Gabon. African Journal of Ecology 31: 181–199.

[17] Turkalo A, Fay JM (1996) Studying forest elephants by direct observation: preliminary results from the Dzanga clearing, Central African Republic. Pachyderm 21: 45–54.

[18] Munshi-South J (2011) Relatedness and demography of African forest elephants: inferences from noninvasive fecal DNA analyses. Journal of Heredity 102: 391–398. 21576286 10.1093/jhered/esr030

[19] Fishlock V, Lee PC, Breuer T (2008) Quantifying forest elephant social structure in Central African bai environment. Pachyderm 44: 17–26.

[20] Momont L (2007) Sélection de l'habitat et organisation sociale de l'éléphant de forêt, *Loxodonta africana cyclotis* (Matschie 1900), au Gabon: Muséum National d'Histoire Naturelle.

[21] White LJT (1994) Biomass of rain-forest mammals in the Lopé Reserve, Gabon. Journal of Animal Ecology 63: 499–512.

[22] White LJT (1995) Factors affecting the duration of elephant dung piles in rain forest in the Lopé Reserve, Gabon. African Journal of Ecology 33: 142–150.

[23] Eggert LS, Eggert JA, Woodruff DS (2003) Estimating population sizes for elusive animals: the forest elephants of Kakum National Park, Ghana. Molecular Ecology 12: 1389–1402. 12755869 10.1046/j.1365-294x.2003.01822.x

[24] Amos W, Whitehead H, Ferrari MJ, Glockner-Ferrari DA, Payne R, et al (1992) Restrictable DNA from sloughed cetacean skin; its potential for use in population analysis. Marine Mammal Science 8: 275–283.

[25] Archie EA, Moss CJ, Alberts SC (2003) Characterization of tetranucleotide microsatellite loci in the African savannah elephant (*Loxodonta africana africana*). Molecular Ecology Notes 3: 244–246.

[26] Eggert LS, Maldonado JE, Fleischer RC (2005) Nucleic acid isolation from ecological samples: animal scat and other associated materials. Molecular Evolution: Producing the Biochemical Data Part B: 73–87. 10.1016/S0076-6879(05)95006-4 15865962

[27] Eggert LS, Ahlering M, Manka SG. Lessons from genetic censuses of forest elephants. In: Olson D, editor; 2008 November, 2007; International Elephant Foundation and Ringling Brothers Barnum and Bailey Center for Elephant Conservation. pp. 157–164.

[28] Comstock KE, Wasser SK, Ostrander EA (2000) Polymorphic microsatellite DNA loci identified in the African elephant (*Loxodonta africana*). Molecular Ecology 9: 1004–1006. 10886668 10.1046/j.1365-294x.2000.00939-8.x

[29] Gobush K, Kerr BEN, Wasser S (2009) Genetic relatedness and disrupted social structure in a poached population of African elephants. Molecular Ecology 18: 722–734. 19175507 10.1111/j.1365-294X.2008.04043.x

[30] Ahlering MA, Maldonado JE, Fleischer RC, Western D, Eggert LS (2012) Fine-scale group structure and demography of African savanna elephants recolonizing lands outside protected areas. Diversity and Distributions 18: 952–961.

[31] Frantz AC, Pope LC, Carpenter PJ, Roper TJ, Wilson GJ, et al (2003) Reliable microsatellite genotyping of the Eurasian badger (*Meles meles*) using faecal DNA. Molecular Ecology 12: 1649–1661. 12755892 10.1046/j.1365-294x.2003.01848.x

[32] Hansen H, Ben-David M, McDonald DB (2008) Effects of genotyping protocols on success and errors in identifying individual river otters (*Lontra canadensis*) from their faeces. Molecular Ecology Resources 8: 282–289. 21585770 10.1111/j.1471-8286.2007.01992.x

[33] Waits LP, Luikart G, Taberlet P (2001) Estimating the probability of identity among genotypes in natural populations: cautions and guidelines. Molecular Ecology 10: 249–256. 11251803 10.1046/j.1365-294x.2001.01185.x

[34] Peakall ROD, Smouse PE (2006) GENALEX 6: genetic analysis in Excel. Population genetic software for teaching and research. Molecular Ecology Notes 6: 288–295. 10.1093/bioinformatics/bts460 PMC3463245 22820204

[35] Archie EA, Moss CJ, Alberts SC (2006) The ties that bind: genetic relatedness predicts the fission and fusion of social groups in wild African elephants. Proceedings of the Royal Society Biological Sciences Series B 273: 513–522. 10.1098/rspb.2005.3361 PMC1560064 16537121

[36] Miller CR, Joyce P, Waits LP (2002) Assessing allelic dropout and genotype reliability using maximum likelihood. Genetics 160: 357–366. 11805071 10.1093/genetics/160.1.357 PMC1461941

[37] McKelvey KS, Schwartz MK (2005) DROPOUT: a program to identify problem loci and samples for noninvasive genetic samples in a capture-mark-recapture framework. Molecular Ecology Notes 5: 716–718.

[38] Munshi-South J, Tchignoumba L, Brown J, Abbondanza N, Maldonado JE, et al (2008) Physiological indicators of stress in African forest elephants (*Loxodonta africana cyclotis*) in relation to petroleum operations in Gabon, Central Africa. Diversity and Distributions 14: 995–1003.

[39] Ahlering MA, Hailer F, Roberts MT, Foley C (2011) A simple and accurate method to sex savannah, forest and asian elephants using noninvasive sampling techniques. Molecular Ecology Resources 11: 831–834. 21635697 10.1111/j.1755-0998.2011.03030.x

[40] Fernando P, Pfrender ME, Encalada SE, Lande R (2000) Mitochondrial DNA variation, phylogeography and population structure of the Asian elephant. Heredity 84: 362–372. 10762406 10.1046/j.1365-2540.2000.00674.x

[41] Eggert LS, Rasner CA, Woodruff DS (2002) The evolution and phylogeography of the African elephant inferred from mitochondrial DNA sequence and nuclear microsatellite markers. Proceedings of the Royal Society of London Series B 269: 1993–2006. 12396498 10.1098/rspb.2002.2070 PMC1691127

[42] Sukumar R (2003) The Living Elephants: Evolutionary Ecology, Behavior, and Conservation. New York: Oxford University Press.

[43] van Oosterhout C, Hutchinson WF, Wills DPM, Shipley P (2004) MICRO-CHECKER: software for identifying and correcting genotyping errors in microsatellite data. Molecular Ecology Notes 4: 535–538.

[44] Raymond M, Rousset F (1995) GENEPOP (version 1.2): population genetics software for exact tests and ecumenicism. Journal of Heredity 86: 248–249.

[45] Queller DC, Goodnight K (1989) Estimating relatedness using genetic markers. Evolution 43: 258–275. 28568555 10.1111/j.1558-5646.1989.tb04226.x

[46] Smouse PE, Peakall R (1999) Spatial autocorrelation analysis of individual multiallele and multilocus genetic structure. Heredity 82: 561–573. 10383677 10.1038/sj.hdy.6885180

[47] Peakall R, Ruibal M, Lindenmayer DB (2003) Spatial autocorrelation analysis offers new insights into gene flow in the Australian bush rat, *Rattus fuscipes* . Evolution 57: 1182–1195. 12836834 10.1111/j.0014-3820.2003.tb00327.x

[48] Van De Casteele T, Galbusera P, Matthysen E (2001) A comparison of microsatellite-based pairwise relatedness estimators. Molecular Ecology 10: 1539–1549. 11412374 10.1046/j.1365-294x.2001.01288.x

[49] Wang J (2011) COANCESTRY: a program for simulating, estimating and analysing relatedness and inbreeding coefficients. Molecular Ecology Resources 11: 141–145. 21429111 10.1111/j.1755-0998.2010.02885.x

[50] Hemelrijk CK (1990) A matrix partial correlation test used in investigations of reciprocity and other social interaction patterns at group level. Journal of Theoretical Biology 143: 405–420.

[51] Duchesne P, ÉTienne C, Bernatchez L (2006) PERM: a computer program to detect structuring factors in social units. Molecular Ecology Notes 6: 965–967.

[52] Borgatti SP, Everett MG, Freeman LC (2002) Ucinet for Windows: software for social network analysis. Harvard, MA: Analytic Technologies.

[53] Borgatti SP (2002) NetDraw software for network visualization. Lexington, KY: Analytic Technologies.

[54] Van Coeverden de Groot P, Putnam A, Erb P, Scott C, Melnick D, et al (2011) Conservation genetics of the black rhinoceros, *Diceros bicornis bicornis*, in Namibia. Conservation Genetics 12: 783–792.

[55] Pope LC, Domingo-Roura X, Erven K, Burke T (2006) Isolation by distance and gene flow in the Eurasian badger (*Meles meles*) at both a local and broad scale. Molecular Ecology 15: 371–386. 16448407 10.1111/j.1365-294X.2005.02815.x

[56] Schuttler SG, Blake S, Eggert LS (2012) Movement patterns and spatial relationships among African forest elephants. Biotropica 44: 445–448.

[57] White LJT (1994) *Sacoglottis gabonensis* fruiting and the seasonal movements of elephants in the Lopé Reserve, Gabon. Journal of Tropical Ecology 10: 121–125.

[58] Maisels F, Strindberg S, Blake S, Wittemyer G, Hart J, et al (2013) Devastating decline of forest elephants in Central Africa. PLoS ONE 8: e59469. 23469289 10.1371/journal.pone.0059469 PMC3587600

[59] Wittemyer G, Okello JBA, Rasmussen H, Arctander P, Nyakaana S, et al (2009) Where sociality and relatedness diverge: the genetic basis for hierarchical social organization in African elephants. Proceedings of the Royal Society B 276: 3513–3521. 19605399 10.1098/rspb.2009.0941 PMC2817196

[60] Wrege PH, Rowland ED, Bout N, Doukaga M (2012) Opening a larger window onto forest elephant ecology. African Journal of Ecology 50: 176–183.

