# Supplementary material for: Fine-Scale Genetic Structure and Cryptic Associations Reveal Evidence of Kin-Based Sociality in the African Forest Elephant
Source: PLoS One. 2014 Feb 5;9(2):e88074. doi: 10.1371/journal.pone.0088074 (PMC3914907; doi:10.1371/journal.pone.0088074)
Supplement: Table S1 — Genetic diversity values for elephants at Lopé National Park, Gabon. Na = allelic diversity, He = expected and Ho = observed heterozygosity. Multiplexes 1 and 4 had an annealing temperature of 60°C, while 2 and 3 were at 58°C. (DOCX) [file pone.0088074.s001.docx]

Table S1. Genetic diversity values for elephants at Lopé National Park, Gabon (N_a_=allelic diversity, H_e_=expected and H_o_=observed heterozygosity). Multiplexes 1 and 4 had an annealing temperature of 60°C, while 2 and 3 were at 58°C.

| **locus** | **N_a_** | **H_e_** | **H_o_** | **multiplex number** |
| --- | --- | --- | --- | --- |
| FH94R | 9 | 0.653 | 0.676 | 2 |
| FH126 | 16 | 0.884 | 0.879 | 2, 3, 4 |
| FH103R | 8 | 0.794 | 0.785 | 2 |
| LaT13R | 20 | 0.917 | 0.905 | 1, 2, 3, 4 |
| FH67 | 8 | 0.728 | 0.729 | 3 |
| FH48R | 13 | 0.832 | 0.829 | 1 |
| LA6R | 10 | 0.723 | 0.750 | 3 |
| FH60R | 12 | 0.850 | 0.867 | 1 |
| FH19R | 15 | 0.898 | 0.910 | 1, 4 |
| FH129 | 12 | 0.861 | 0.870 | 4 |
| Mean | 12 | 0.841 | 0.820 |  |
| SD | 3.9 | 0.087 | 0.081 |  |
