# Supplementary material for: Fine-Scale Genetic Structure and Cryptic Associations Reveal Evidence of Kin-Based Sociality in the African Forest Elephant
Source: PLoS One. 2014 Feb 5;9(2):e88074. doi: 10.1371/journal.pone.0088074 (PMC3914907; doi:10.1371/journal.pone.0088074)
Supplement: Table S2 — Group composition, mitochondrial haplotypes, average pairwise relatedness (R) and 95% confidence intervals (CI) within groups. A–adult, J–juvenile, U–unknown age category, F–female, and M–male. (DOCX) [file pone.0088074.s002.docx]

**Table S2**. **Group composition, mitochondrial haplotypes, average pairwise relatedness (*R*) and 95% confidence intervals (CI) within groups.** A–adult, J–juvenile, U–unknown age category, F-female, and M – male.

| **group** | **n** | **group composition** | **haplotype** | **R** | **CI** |
| --- | --- | --- | --- | --- | --- |
| B0108 | 2 | AF, JM | Lope5 | 0.230 | 0.371 |
| B0110 | 3 | AF, 2 UF | Lope1 | 0.224 | 0.144 |
| B0208 | 2 | AF, JM | Lope7 | -0.156 | 0.177 |
| B0310 | 2 | 2 AF | Lope9 | 0.491 | 0.205 |
| B0608 | 2 | AF, UF | Lope7 | 0.319 | 0.260 |
| B0610 | 3 | 2 AF, JM | Lope7 | 0.101 | 0.217 |
| B0708 | 3 | 2 AF, JF | Lope1 | 0.368 | 0.180 |
| B0908 | 2 | AF, JF | Lope7 | 0.499 | 0.225 |
| B1210 | 3 | 2 AF, JF | Lope7, Lope9 | 0.257 | 0.281 |
| B1310 | 4 | 3 AF, JF | Lope4 | 0.255 | 0.183 |
| B1508 | 2 | AF, JM | Lope6 | 0.439 | 0.106 |
| B1510 | 2 | AF, JM | Lope7 | 0.350 | 0.175 |
| B1710 | 3 | AM, UF, UM | Lope4, Lope7 | 0.082 | 0.166 |
| B1810 | 4 | 2 AF, 2 JF | Lope7 | 0.281 | 0.172 |
| B1908 | 3 | 2 AF, JM | Lope4 | 0.308 | 0.213 |
| B1910 | 2 | AF, JM | Lope7 | 0.404 | 0.145 |
| B2008 | 2 | AF, JM | Lope7 | 0.351 | 0.175 |
| B3810 | 2 | 2 AF | Lope7 | -0.134 | 0.204 |
| B2010 | 5 | 3 AF, JF, JM | Lope7 | 0.074 | 0.108 |
| B2108 | 6 | 3 AF, AM, JF, JM | Lope7 | 0.031 | 0.054 |
| B2110 | 2 | AF, JM | Lope7 | 0.343 | 0.174 |
| B2210 | 2 | AF, JM | Lope7 | 0.526 | 0.202 |
| B2310 | 4 | AF, 2 JF, JM | Lope3, Lope7 | 0.078 | 0.154 |
| B2410 | 2 | AF, JM | Lope7 | 0.350 | 0.175 |
| B2510 | 2 | AF, JF | Lope3, Lope7 | -0.147 | 0.316 |
| B2610 | 2 | 2 AF | Lope3 | 0.271 | 0.300 |
| B2710 | 2 | 2 AF | Lope5 | 0.118 | 0.309 |
| B2810 | 2 | AF, JF | Lope3 | 0.517 | 0.202 |
| B2910 | 3 | AF, JF, JM | Lope7 | 0.394 | 0.145 |
| B3010 | 3 | 2 AF, JF | Lope7 | 0.160 | 0.146 |
| B3110 | 2 | AF, JM | Lope7 | 0.499 | 0.233 |
| B3310 | 2 | AF, JF | Lope7 | 0.423 | 0.104 |
| B3410 | 2 | 2 AF | Lope5 | 0.506 | 0.237 |
| B3510 | 2 | 2 AF | Lope7 | 0.093 | 0.220 |
| B3610 | 2 | AF, JM | Lope7 | 0.422 | 0.142 |
| **group** | **n** | **group composition** | **haplotype** | **R** | **CI** |
| S0110 | 2 | AF, JF | Lope7 | 0.384 | 0.178 |
| S0308 | 2 | AF, JM | Lope7 | 0.499 | 0.233 |
| S0508 | 2 | 2 UF | Lope1, Lope9 | 0.006 | 0.374 |
| S0510 | 4 | AF, UF, UM, JF, | Lope7 | 0.265 | 0.138 |
| S0608 | 4 | 2 AF, 2 JF | Lope3 | 0.149 | 0.102 |
| S0708 | 2 | AF, JM | Lope9 | 0.222 | 0.436 |
| S0710 | 2 | JF, UF | Lope3 | -0.016 | 0.393 |
| S0808 | 2 | AF, UF | Lope3, Lope7 | 0.019 | 0.327 |
| S1008 | 2 | AF, JF | Lope7 | 0.382 | 0.177 |
| S1108 | 2 | 2 AF | Lope5 | 0.304 | 0.361 |
| S1110 | 2 | AF, JM | Lope7 | 0.241 | 0.284 |
| S1308 | 2 | AF, JM | Lope7 | 0.422 | 0.142 |
| S1310 | 2 | AF, UF | Lope4, Lope9 | 0.087 | 0.278 |
| S1408 | 2 | AF, JF | Lope7 | 0.466 | 0.187 |
| S1410 | 3 | AF, 2 JM | Lope7 | 0.367 | 0.186 |
| S1508 | 2 | AF, JF | Lope3 | 0.517 | 0.202 |
| S1510 | 2 | AF, JF | Lope7 | 0.384 | 0.178 |
| S1610 | 2 | AM, AF | Lope1, Lope6 | -0.273 | 0.208 |
| S1708 | 2 | AF, JF | Lope5 | 0.533 | 0.404 |
| S1808 | 2 | AF, UM | Lope7 | 0.288 | 0.299 |
| S1908 | 2 | 2 AF | Lope7 | 0.493 | 0.325 |
| S1910 | 2 | 2 JM | Lope1 | 0.256 | 0.474 |
| S2008 | 3 | AF, AM, JF, | Lope5, Lope7 | 0.070 | 0.109 |
| S2010 | 2 | 2 AF | Lope1 | 0.273 | 0.415 |
| S2108 | 2 | 2 AF | Lope7 | 0.376 | 0.272 |
| S2210 | 5 | 3 AF, AM, UM | Lope7 | 0.236 | 0.211 |
| S2308 | 4 | 3 AF, JF | Lope3 | 0.368 | 0.148 |
| S2408 | 2 | 2 AF | Lope3, Lope7 | -0.172 | 0.257 |
| S2508 | 3 | 2 AF, 1JF | Lope1, Lope3, Lope7 | -0.064 | 0.148 |
| S2610 | 5 | 3 AF, 2 JF | Lope9 | 0.358 | 0.156 |
| S2708 | 2 | AF, JM | Lope5 | 0.272 | 0.125 |
| S2710 | 3 | AF, JM | Lope7 | 0.116 | 0.394 |
| S2808 | 3 | 2 AF, JM | Lope7 | 0.142 | 0.178 |
| S2810 | 2 | AF, AM | Lope1, Lope7 | 0.149 | 0.300 |
| S2910 | 3 | AF, JF, JM | Lope7 | 0.394 | 0.145 |
| Mean |  |  |  | 0.255 |  |
